# Acute two-photon imaging of the neurovascular unit in the cortex of active mice

**DOI:** 10.3389/fncel.2015.00011

**Published:** 2015-02-05

**Authors:** Cam Ha T. Tran, Grant R. Gordon

**Affiliations:** Hotchkiss Brain Institute, Department of Physiology and Pharmacology, Cumming School of Medicine, University of CalgaryCalgary, AB, Canada

**Keywords:** two-photon, acute, non-anesthetised, fully awake, cranial window, mice, neurovascular unit, astrocyte

## Abstract

*In vivo* two-photon scanning fluorescence imaging is a powerful technique to observe physiological processes from the millimeter to the micron scale in the intact animal. In neuroscience research, a common approach is to install an acute cranial window and head bar to explore neocortical function under anesthesia before inflammation peaks from the surgery. However, there are few detailed acute protocols for head-restrained and fully awake animal imaging of the neurovascular unit during activity. This is because acutely performed awake experiments are typically untenable when the animal is naïve to the imaging apparatus. Here we detail a method that achieves acute, deep-tissue two-photon imaging of neocortical astrocytes and microvasculature in behaving mice. A week prior to experimentation, implantation of the head bar alone allows mice to train for head-immobilization on an easy-to-learn air-supported ball treadmill. Following just two brief familiarization sessions to the treadmill on separate days, an acute cranial window can subsequently be installed for immediate imaging. We demonstrate how running and whisking data can be captured simultaneously with two-photon fluorescence signals with acceptable movement artifacts during active motion. We also show possible applications of this technique by (1) monitoring dynamic changes to microvascular diameter and red blood cells in response to vibrissa sensory stimulation, (2) examining responses of the cerebral microcirculation to the systemic delivery of pharmacological agents using a tail artery cannula during awake imaging, and (3) measuring Ca^2+^ signals from synthetic and genetically encoded Ca^2+^ indicators in astrocytes. This method will facilitate acute two-photon fluorescence imaging in awake, active mice and help link cellular events within the neurovascular unit to behavior.

## Introduction

Two-photon scanning fluorescence microscopy has greatly facilitated the study of numerous cellular and physiological processes in intact tissue. The study of the neurovascular unit as it pertains to local brain blood flow control, is a notable example of how two-photon microscopy has advanced our understanding of the architecture of the cerebral microcirculation (Kleinfeld et al., 1998; Blinder et al., 2010) and how cellular brain activity is functionally linked to control microvascular blood flow, a process termed neurovascular coupling (Takano et al., 2006; Chuquet et al., 2007; Winship et al., 2007; Helmchen and Kleinfeld, 2008; Schummers et al., 2008; Wang et al., 2009; Kuga et al., 2011; McCaslin et al., 2011; Shen et al., 2012; Shih et al., 2012b; Thrane et al., 2012; Ding et al., 2013; Lind et al., 2013; Nizar et al., 2013). Two-photon imaging allows deep tissue observations and is capable of monitoring subcellular changes in Ca^2+^ (an essential second messenger in brain to blood flow coupling) and several parameters of blood flow such as vessel diameter, red blood cell velocity and red blood cell flux (Kleinfeld et al., 1998; Takano et al., 2006; Stefanovic et al., 2008; Shih et al., 2012b; Nizar et al., 2013).

In order to visualize brain cells and blood flow dynamics in the neocortex, a widely adopted method is to perform an acute craniectomy over a cortical region of interest and then mount cover glass that either fully or partially seals the area creating a window. Full bone and dura removal enables the deepest imaging or the capture of faint fluorescence signals such as that which arises from intrinsic NAD(P)H (Kasischke et al., 2011). By keeping the animal under anesthesia after window implantation, two-photon imaging can be conducted right after surgery. Immediate imaging is a critical aspect of this procedure because inflammation remains low at this time, peaking several days after the craniotomy (Xu et al., 2007; Holtmaat et al., 2009), and acutely administered dexamethasone is used to control early inflammatory processes (Holtmaat et al., 2009; Nimmerjahn, 2012; Zariwala et al., 2012; Johnston et al., 2013). Furthermore, reactive astrocytes and activated microglia become apparent 24–48 h after surgery (Xu et al., 2007; Grutzendler et al., 2011) and these changes take approximately 4 weeks to subside (Xu et al., 2007; Holtmaat et al., 2009). Thus, imaging should either take place immediately, or 1 month after surgery. Having to wait 4 weeks, with the additional risks of the window clouding or growing over, is a key determinant in the continued and wide spread use of acute windows.

While anesthetics eliminate animal pain, reduce movement artifacts during imaging, and provide a controlled experimental platform, anesthetics have dramatic effects on neural activity (Kebabian et al., 1975; Lenox et al., 1979; Hentschke et al., 2005), astrocyte Ca^2+^ (Thrane et al., 2012), vessel contractility (Pisauro et al., 2013) and oxygen consumption (Lei et al., 2001). Undoubtedly, these side effects are significant confounds for the study of the neurovascular unit, as well as many others research fields. Therefore, there is a clear need for cellular level imaging experiments in awake, behaving animals, yet there are few detailed acute imaging methodologies for head-restrained, fully awake animals (Dombeck et al., 2007). Head restraint is necessary for standard two-photon imaging so that detailed cellular level data can be achieved, however, head immobilization is a significant stressor for animals that are naïve to the condition (Schwarz et al., 2010). Furthermore, there are limited techniques to manipulate the brain and microvasculature when employing a fully sealed cranial window for awake imaging. Although, partially open windows permit the delivery of common pharmacological agents, these windows may increase movement artifacts during awake imaging. To increase the breadth of possible applications toward the study of neurovascular coupling in awake animals, and to better optimize mouse behaviors during head-restrained two-photon imaging, additional approaches are needed.

Here we provide a detailed method that enables acute awake two-photon imaging of neocortical astrocytes and the microvasculature of active mice. Our strategy involves (1) a separation from head bar implantation to the creation of an acute cranial window to permit training for the experimental setup before imaging, (2) the design/adaptation of an easy to learn air-supported spherical treadmill for awake head-restrained experiments and (3) a brief yet effective training regime for head-restraint and vibrissa stimulation. This methodology helps reduce the appearance of stress behaviors and enables deep, subcellular measurements of brain cells and the microcirculation with acceptable movement artifacts. When combined with near infrared videography, behaviors such as whisking, grooming, resting, and running can be captured simultaneously with two-photon imaging. Additionally, we provide a method for manipulating the brain and microvasculature by perfusing pharmacological agents through a tail cannula during awake imaging. When included with a dye, intraluminal fluorescence approximates the arrival of the pharmacological agent that can be used to understand the timing of brain and vascular effects. Finally, we demonstrate the measurement of subcellular Ca^2+^ signals from synthetic and genetically encoded Ca^2+^ indicators in astrocytes, cells that have extensive endfeet covering of the microvascular wall and that regulate arteriole diameter (Zonta et al., 2003). This method will facilitate acute, deep tissue two-photon fluorescence imaging of the neurovascular unit in active behaving mice.

## General methods

### Animals

All animal procedures are detailed on institutional protocol M11032 and were approved by the Animal Care and Use Committee of the University of Calgary. All studies were performed on male C57Bl/6 mice or male GLAST-Cre-ERT × LSL-GCaMP3 mice (Jax Lab #012586 and #014538) between P30 to P60. For GCaMP3 experiments, we initiated our acute awake imaging protocol 3 weeks after three consecutive tamoxifen injections (100 mg/kg, stock of 10 mg/mL in corn oil, Sigma, St. Louise, MO, USA). All surgeries used standard aseptic procedures. Animals were anesthetized with isoflurane (Pharmaceutical Partners of Canada Inc., Richmond Hill ON) with 5% used for induction and 1–1.5% for maintenance during surgery at the level of reflex suppression. Mice were mounted in a stereotaxic frame (Stoelting, Harvard Apparatus Canada, Saint-Laurent QC). Animals were kept on a heating blanket with a feedback rectal probe to maintain body temperature at 37°C (Homeothermic blanket monitoring system, Harvard Apparatus).

### Two-photon data acquisition and analysis

A custom *in vivo* two-photon microscope was fed by a broadly tunable Ti:sapphire laser (Coherent Chameleon, Ultra II, ~4 W avg power, 670–1080 nm, ~80 MHz, 140 fs pulse width). The microscope is controlled by open-source ScanImage software (https://openwiki.janelia.org/) that runs in MATLAB and uses acquisition hardware from National Instruments. We use a Nikon 16X, 0.8NA, 3 mm WD objective lens for a large field of view, as well as a Zeiss 40X, 1.0NA, 2.5 mm WD objective lens for a higher numerical aperture. We use 5 mm galvanometric mirrors for scanning (Cambridge Technology Inc., MA, USA) and GaAsP PMTs for fluorescence light detection (Hamamatsu, Japan). We excite Rhod2-AM (Biotium, Hayward, CA, USA) at 850 nm and GCaMP3 at 940 nm. Green fluorescence signals were collected through a 50 nm bandpass filter centered at 525 nm. Orange/Red fluorescence signals were collected through a 70 nm bandpass filter centered at 605 nm (Chroma Technology, Bellows Falls, VT, USA). Vasomotor responses of the pial and penetrating arterioles as well as astrocytic Ca^2+^ activities were monitored using either xy raster scanning (0.98–7.8 Hz) or line scanning (500 Hz). RBCs within capillaries were monitored by placing a scanning path along the longitudinal axis of the vessel as previously described (Kleinfeld et al., 1998; Shih et al., 2012a). RBC velocity was calculated by v = x/Δt where x is the distance and Δt is the time of RBC travel. RBC flux was calculated as the number of cells transiting per second in single file along the capillary (Kleinfeld et al., 1998). All data was processed using ImageJ. Movement artifacts in the xy plane were corrected for using the template_matching plugin. The 3D viewer plugin was used to perform three-dimensional rendering of the microvascular network. Ca^2+^ responses were calculated as ΔF/F = (F_t_ − Frest)/Frest, where F_t_ was the measured fluorescence signal at any given time point and Frest is the average baseline fluorescence.

## Protocol methods and results

### Summary

**Figure 1:**
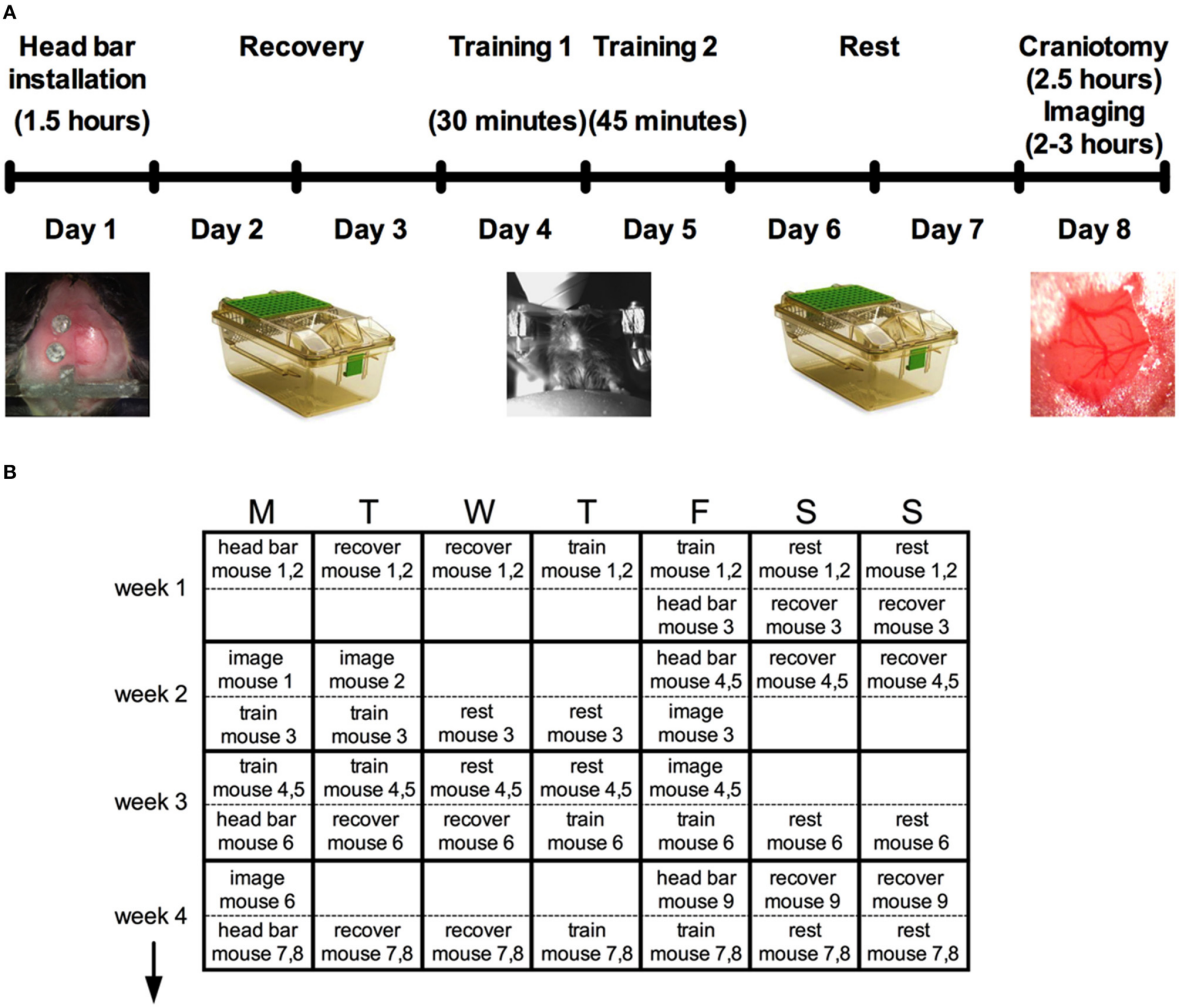
**Protocol summary for acute awake two-photon imaging in active mice**. **(A)** Schematic timeline highlighting six main steps and the approximate time required for each: head bar installation, recovery, training, and the craniotomy before imaging. **(B)** A typical schedule over a period of 4 weeks to manage multiple mice through the protocol.

On day one, minor surgery is performed to implant a head bar and prepare the skull for a future acute cranial window. After resting for 2 days, a brief 30 min training session to head restraint is conducted, and again for 45 min on the following day using an easy-to-learn passive spherical treadmill. During these sessions, we also train the animal to accept vibrissa stimulation using air puff. Following two more rest days an acute cranial window is installed for immediate two-photon imaging (Figure 1A).

Head bar installation and training prior to creating the cranial window enabled the animals to learn the treadmill and the experimental environment so that behaviors such as struggling, freezing and squeaking were largely eliminated during imaging. While the entire protocol per animal is stretched out over 8 days (Figure 1A), the total time working on a single animal (including all surgeries, training, transferring and imaging) is under 8 h, permitting a regular productive schedule. For instance, we structure our experiments based on a 5-day workweek, leaving animal recovery or resting over the weekend. We provide a theoretical schedule for how multiple mice can be processed over a 4-week period (Figure 1B). The throughput can be increased from what is suggested by working with more mice simultaneously. However, the time for analysis is significant and can easily fill the open periods of the schedule once good data has been collected.

### Head bar installation

**Figure 2:**
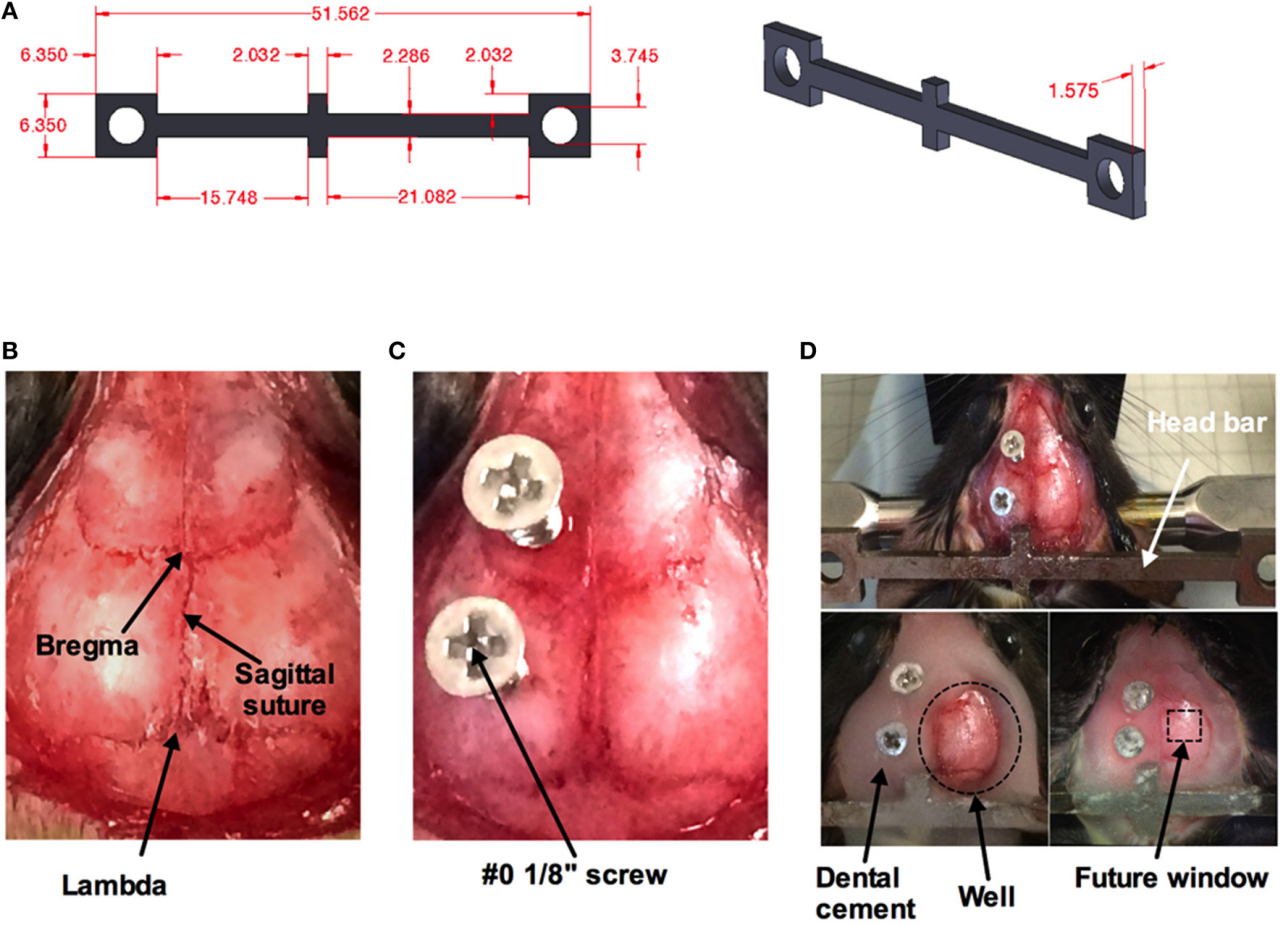
**Surgical procedure for head bar installation**. **(A)** Measurements (in mm) for custom made mouse head bar. **(B)** An image of the top of a mouse head with the skin and the thin layer of periosteum removed by scalpel blade revealing bregma, lambda, and the sagittal suture. **(C)** Two #0 1/8” screws are superficially screwed down (about one rotation) to the frontal and parietal bone of the contralateral side. A thin layer of cyanoacrylate has been applied over the entire skull. **(D)** A custom-made head bar is placed over the inter-parietal bone and covered with a thin layer of cyanoacrylate (top). Dental cement is applied to the entire skull except where the window will be placed. Dotted circle indicates the well for a water-dipping objective (bottom left). Dotted square depicts where the cranial window will be created in 1 week's time (bottom right).

A minor surgery is performed to install the head bar 1 week before the imaging session (Figure 2). The head bar is an integral part in ensuring the animal's head is securely immobilized during imaging. The bar is custom-made and a modification to a design provided by the Kleinfeld laboratory (http://neurophysics.ucsd.edu/lab_hardware.php). The modification extends the length of the long arm (Figure 2A), which optimizes access of the objective lens to an off-axis location such as the barrel cortex when using a rotatable *in vivo* microscope. Prior to the surgery, all surgical instruments are sterilized using a dry sterilizer (Germinator 500, Braintree Scientific Inc., Braintree, MA, USA). Once the animal is anesthetized, a single dose of buprenorphine (0.05 mg/kg, controlled drug supplied by institutional Animal Resource Centre) and enrofloxacin (2.5 mg/kg, Bayer Health Care, Toronto ON Canada) was injected subcutaneously in the back region of the animal prior to surgery. Sterile ophthalmic gel (Dechra – Aventix Animal Health, Burlington ON Canada) is applied to the eyes to prevent dehydration and irritation. The following procedures are modified from previous works (Holtmaat et al., 2009; Shih et al., 2012b) to suit our need to train the animals before acute imaging. The fur on the top of the head is shaved off. The skin is cleaned with three alternating swabs of betadine and alcohol. An incision of about 3 cm down the midline of the skull is performed using a scalpel. The start of the incision is from between the eyes and moving past the ears toward the interparietal bone. The two flaps of skin over the skull covering both hemispheres are removed using a pair of small surgical scissors. Any bleeding is controlled by applying pressure with eye spears (Beaver Visitec, Waltham, MA, USA). The thin layer of periosteum is gently scrapped off using a scalpel blade (Figure 2B). Removing this thin layer will enhance the bonding of the cyanoacrylate (3M Vetbond™, 3M Animal Care Products, St. Paul, MN, USA) to the skull. Two small holes are made using a dental drill (Microtorque II, Harvard Apparatus) with a small bit burr size (0.019 in diameter) over the frontal bone and parietal bone of the contralateral side to the future cranial window. Two self-tapping screws (Amazon.com, size #0, length 1/8”) are placed in the two holes and screwed down until they securely grab the bone but do not impact the brain tissue (turn the screw about 1 rotation) (Figure 2C). The screws, when combined with the cement (later step), increase the connection strength of the entire head-bar assembly to the skull. Cyanoacrylate is then applied to the entire skull. Lactated Ringer's (in g/100 mL: 0.6 NaCl; 0.31 C3H5NaO3; 0.03 KCl; 0.02 CaCl2^*^2H2O) is applied regularly to reduce heat, and the depth of the drill should be well controlled to avoid penetrating through the bone and damaging the cortical tissue. Next the head bar is positioned over the interparietal bone and glued down with cyanoacrylate (Figure 2D top panel), taking care that it is level both laterally and rostro-caudally. Once the cyanoacrylate is dried, dental cement (Ortho Jet Powder and Liquid, Lang Dental Manufacturing Co., Wheeling IL USA) is then applied, covering the head bar and screws, but not over the region where the window will be implanted in 1 week's time. Furthermore, a well must be progressively built up using the cement, such that the entire circumference around the future window will hold solution for a water dipping objective lens. This is most critical along the temporal aspect of the skull (Figure 2D bottom panels). The animal is returned to its cage once the dental cement has cured. Monitor the animal over the next 2 days. Abnormal behaviors such as lack of movement, grooming etc. indicate that the animal is experiencing pain, which would require additional doses of buprenorphine as needed. After 2 days of recovery, the animal is to start training for head restraint on the experimental apparatus.

### Training and behavior monitoring

**Figure 3:**
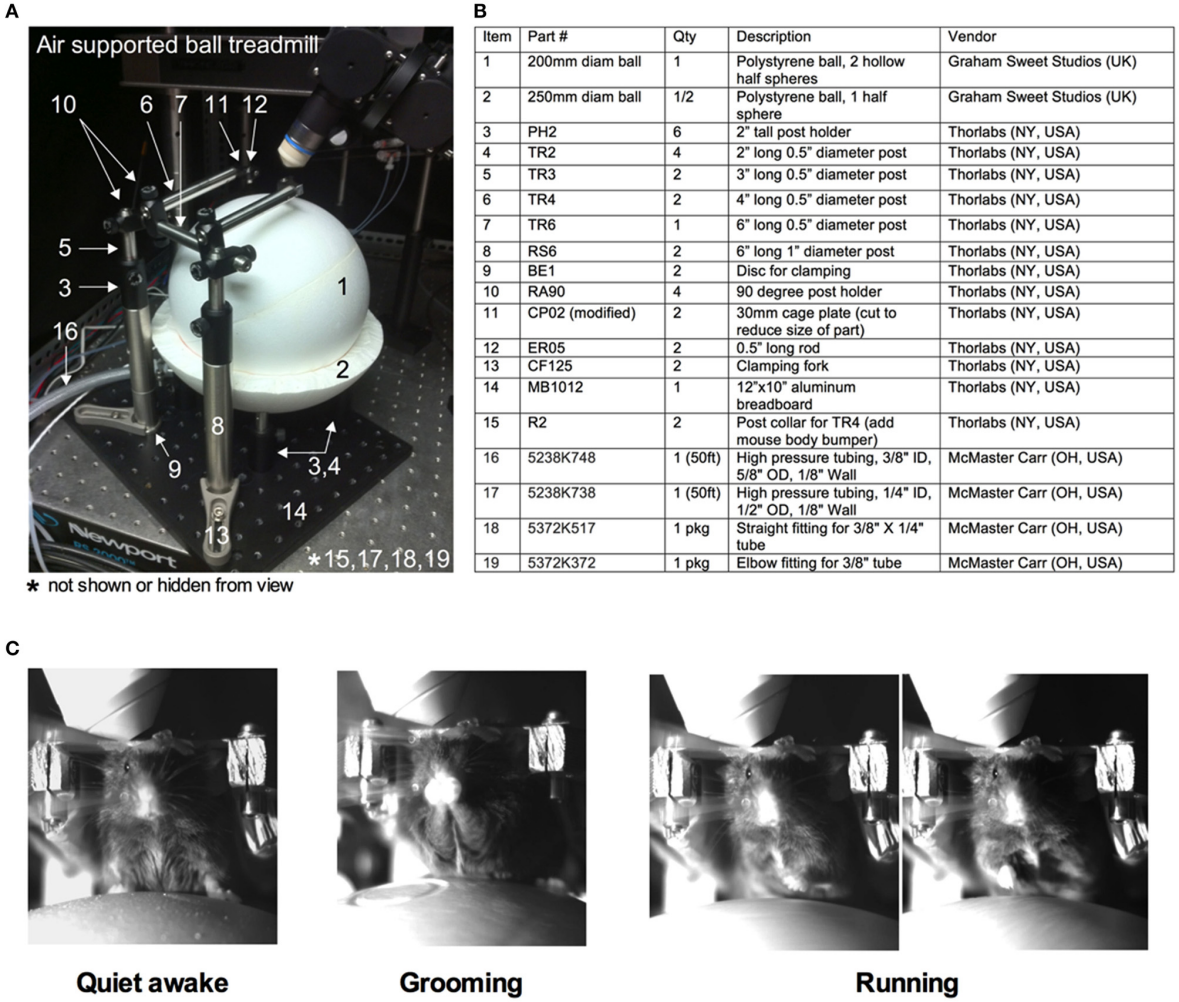
**Training of a mouse for head restraint on an air-supported ball treadmill**. **(A)** Image of the entire air supported ball treadmill apparatus. **(B)** Table listing all the parts. Numbers correspond to those in **(A)**. **(C)** Images captured by a near infrared camera depicting different mouse behaviors while on the treadmill during head immobilization: quiet-awake state (left), grooming (middle), and running (right).

Installation of the head bar without the craniotomy allows training of the animal on the experimental apparatus before acute imaging. The air-supported treadmill is a key component of the success of fully awake, head-restrained imaging (Dombeck et al., 2007) (Figures 3A,B). We float the ball using the least amount of air pressure possible from a standard laboratory tap, to achieve effortless movement of the sphere by hand and by the mouse. Mice immediately begin running on the floating ball. At first, running is erratic and rapid, but as mice learn how to control the ball, particular directions become more consistent and running becomes smooth.

In the training sessions, the animal is exposed to conditions similar to during imaging. The first training session lasts for 30 min. The animal is gently lifted from the cage and placed on the spherical treadmill. The head bar is quickly secured to the platform (modified CP02 plates plus ER05 rods, Thorlabs, Newton NJ USA) with two screws (4–40 screws, McMaster Carr, Aurora, OH, Canada). The treadmill is then slid into positioned under the microscope. The animal is allowed to run on the ball under 780 nm light (M780L3 LED, Thor labs) without interference for 15 min. The animal is then exposed to whisker stimulation. We puff air using a picospritzer III (General Valve Corp., Fairfield, USA) onto the contralateral vibrissa field every minute for 5 s during training. We use the least air pressure that visibly deflects the vibrissae. Alternative sensory stimulations are possible given an appropriate cortical window and given the confines of the experimental apparatus. A similar protocol is repeated for the second 45 min training session on the next day incorporating the same 15 min initial familiarization without air puff. While on the spherical treadmill, the animal exhibits a number of observable behaviors such as being quiet, grooming or running (Figure 3C). After each training session, the animal is returned to the home cage.

### Tail artery cannulation (optional)

Once the animal is trained it can be prepared for acute two-photon imaging. To visualize and quantify changes in microvasculature, a typical method is to perform tail vein injection immediately before imaging with a large molecular weight luminal dye such as fluorescein isothiocyanate-dextran (FITC-dextran, MW 2,000,000) or rhodamine B isothiocyanate-dextran (Rhod-dextran, MW 70,000) (Sigma) (Schaffer et al., 2006; Helmchen and Kleinfeld, 2008; Kerr and Nimmerjahn, 2012). However, tail artery cannulation, rather than tail vein injection, provides potential benefits in addition to standard vascular dye loading. These include: (1) monitoring blood pressure before, during or after an experiment by connection to a blood pressure transducer (such as BIOPAC Systems Inc. Goleta, CA, USA), or (2) delivery of common pharmacological agents (or other desired compounds) systemically during the imaging experiment. The approximate arrival of the agent of interest to the brain microcirculation can be visualized with an aid of a luminal fluorescent dye added to the solution. Here, we provide a brief protocol for tail artery cannulation prior to the craniotomy, with a later example of its application for awake two-photon imaging microcirculation experiments.

**Figure 4:**
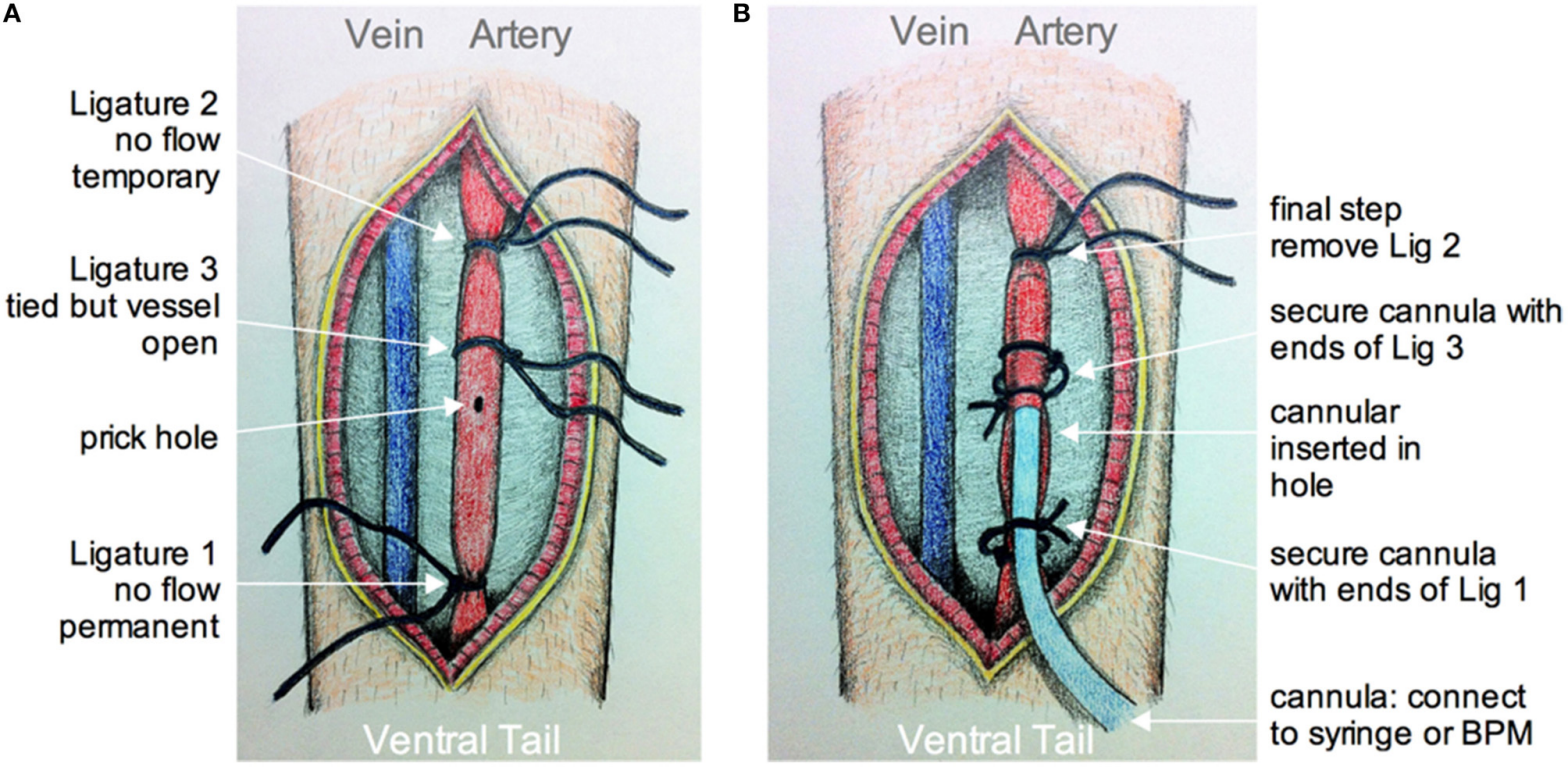
**Schematic of tail artery cannulation**. **(A)** Drawing showing the opened ventral surface of the tail with exposed tail vein and artery. Ligatures are numbered in the order that they are added and tied around the artery. Ligatures 1 and 2, as apposed to 3, tighten around the vessel to prevent flow. After all ligatures are installed, a small hole is made by pricking the artery with a 30G needle. **(B)** Drawing showing the cannula inserted into the hole and through ligature 3. The cannula is secured using the loose ends of ligatures 1 and 3. The final step is to remove ligature 2 so that fluid from the cannula can be fed into an unobstructed artery. The cannula is connected to a syringe and pump, and/or a blood pressure monitor (BPM).

The animal is anesthetized using isoflurane as above. Dexamethasone 21-phosphate disodium salt (0.02 ml at 4 mg/ml; ~2 μg/g, Sigma) is injected intramuscularly to reduce the degree of edema and inflammation (Holtmaat et al., 2009; Drew et al., 2010). Buprenorphine is given through a subcutaneous injection to provide analgesia. The animal is placed on its back so that the ventral side of the tail is facing up, which provides access to the large tail artery (arteria caudalis mediana). The hair over the region of interest is shaved off using a scalpel. A 2 cm longitudinal incision in the skin on the ventral side of the tail is made from a position approximately 2 cm from the base of the tail. The tail artery can be bluntly dissected away from all membrane and connective tissues using forceps and scissors. The artery can then be clamped after lifting the vessel and ligating distally with suture thread (ligature 1, Figure 4A). Next, a second, temporary ligature is added about 1.5 cm proximally to ligature 1 to prevent blood from flowing to the site of cannulation. A third suture thread, which does not pinch the artery closed, is tied in between ligatures 1 and 2. Once in place, a small hole is made into the vascular wall using a 30 gauge needle (Figure 4A). The tapered end of pre-pulled polyethylene tubing PE10 (ID 0.28 mm, OD 0.61 mm, Intramedic™ Becton Dickenson & Company, Sparks MD USA) filled with 100 IU/ml sodium heparin (LEO Pharma Inc.) in lactated Ringer's is then inserted through the hole and pushed forward about 1 cm (Figure 4B). The cannula is then secured inside the tail artery by tying off the middle suture around both vessel and cannula using the loose ends of the suture thread. The proximal temporary ligature is then untied to allow blood to flow into the cannula, indicating a successful cannulation. The cannula is further kept in place by wrapping and tying off the distal ligature. A touch of cyanoacrylate applied to the sutures completes cannula fastening. The cut is then sutured closed and sealed with cyanoacrylate. It can also be dressed with surgical tape. A drop of local anesthetic such as lidocane is applied to help control tail pain during imaging. The free end of the polyethylene tubing from the cannula is connected to a three-way stopcock that enables access to a pressure transducer (optional, not shown), and a syringe with either vascular labeling dye alone, or the dye plus a pharmacological agent of interest.

### Craniotomy

**Figure 5:**
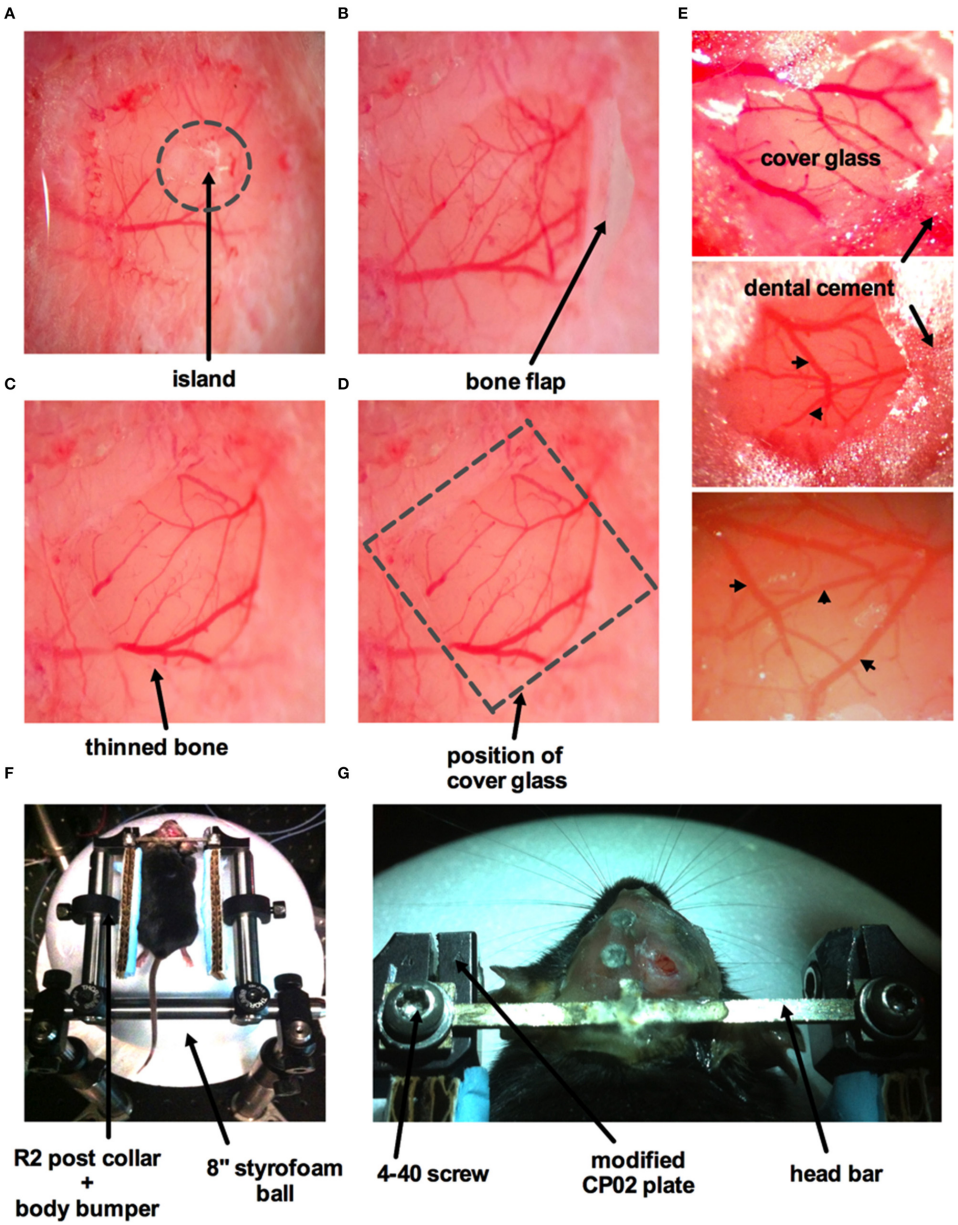
**Surgical procedure for craniotomy with dura removal**. **(A)** Image of part of the skull over the barrel cortex showing a circular groove formed by thinning of the bone creating an island in the middle. **(B)** The island has been lifted off with a bone flap still attached. **(C)** Image of an open window with both bone and dura removed. The arrow points to a band of thinned bone around the circumference of the window. **(D)** Superficial vessels are clearly visible when both bone and dura are removed. Dotted square indicates where the cover glass (#0 thickness) will be placed, same image as **(C)**. **(E)** Three images displaying different cranial windows from three animals. The cover glass is covering the brain and is supported by the thinned bone. Dental cement is applied to the bone and part of the cover glass to fully seal off the cranial window. Arrowheads indicate surface vasculature. **(F)** Mouse placed on the air-supported ball treadmill with its head immobilized immediately after surgery. R2 post collars and attached body bumpers (piece of cardboard with a glued strip of blue surgical pad) are positioned to prevent the animal from swinging sideways once awake, but without obstructing or interfering with the feet. **(G)** Zoomed in view of the custom-made head bar fastened to the modified CP02 plate by 4–40 screws.

This method is adapted from previously published protocols (Mostany and Portera-Cailliau, 2008; Holtmaat et al., 2009; Muniak et al., 2012; Shih et al., 2012b). An anesthetized animal is positioned onto its abdomen and placed in a stereotaxic frame. A 3 × 3 mm square window over the region of interest (i.e., primary somatosensory cortex) is drawn using a fine tip marker. Begin thinning the skull around the perimeter of the square using a dental drill (Microtorque II 35,000 rpm, Harvard Apparatus) on medium speed (~20,000 rpm) with a drill burr size of 0.031 inches in diameter. This step should be performed in short intervals applying as little pressure as possible to the skull surface. After each brief drilling sweep, use an air duster to spray away any debris. Apply cool artificial cerebrospinal fluid (ACSF in mM: 126 NaCl; 2.5 KCl; 25 NaHCO3 1.5 CaCl2^*^2H2O; 1.2 MgCl2^*^6H2O; 1.25 NaH2PO4^*^H2O; 10 Glucose) onto the window to avoid heating the dura mater and brain tissue. A bone island will be formed as the surrounding area of the skull is thinned down (Figure 5A). The vascular network on the surface of the cortex should be clearly visible when the bone is thinned enough. A crack of the spongy bone is typically exposed on the side of the groove once the bone is thinned. Insert one tip of a fine forceps or a micro point horizontally into the edge and then begin lifting the entire island off the skull (Figure 5B). ACSF is applied liberally to facilitate the removal of the bone. Some superficial capillaries of the dura mater can tear as the bone is removed, causing bleeding. If bleeding does not stop automatically, gelfoam that has been soaked in ACSF can be applied on top of the dura mater. After the bleeding has stopped, proceed with removal of the dura mater. This step requires extreme care, as the dura mater is thin and close to the surface of the cortex. A small incision can be made into the dura mater using a 30 gauge needle avoiding perforating the cortical surface. If possible, the incision should be made at the edge of the window where the fewest vessels are present. Micro points and fine forceps can be used to lift the dura mater up and away from the surface of the cortex. Slowly tear the dura mater along the midline of the window and then again along the second midline perpendicular to the first. Pull the flaps of the dura mater toward the edge of the window and flip them on top of the bone. If bleeding occurs during this process, it should be controlled by continuous flushing with ACSF, pre-soaked gelfoam and/or by the use of eye spears. Importantly, the surface of the cortex should never be left to dry. Once the dura mater is removed, an open window surrounded by thinned bone will be achieved (Figure 5C).

#### Synthetic Ca^2+^ indicator loading (optional)

To load astrocytes with a Ca^2+^ indicator, we bulk load Rhod2-AM by applying a Rhod2-AM solution to the exposed surface of the brain for 45 min. To make the solution, first 1 mg of Rhod2-AM is dissolved in 100 μl of DMSO plus 20 μl of a 20% pluronic acid-DMSO solution to create a stock (stored at −20°C). A 6 μl aliquot of the Rhod2-AM stock solution was added to 2994 μl of ACSF to yield a Rhod2-AM concentration of 15 μM. When applied to the brain, the Rhod2-AM solution should be replenished frequently as the solution may evaporate over time or drip off the skull. After the incubation period, excess Rhod2-AM is washed off with ACSF for 10 min. Micro-pipette injection of Ca^2+^ indicator dyes directly into the tissue can also be performed (Winship and Murphy, 2008; Lecoq et al., 2009; Takata et al., 2011).

To finish the window, a drop of ACSF is applied to the surface of the cortex and a pre-cut piece of cover glass (thickness #0) that is slightly larger than the area of exposed brain tissue is placed over the exposed brain. The edges of the cover glass should rest on the thinned bone surrounding the exposed tissue (Figure 5D). Cyanoacrylate is then applied around the edge of the cover glass to fully seal the cranial window. After the cyanoacrylate is dried, dental cement is then applied around the edges of the cover glass to finish the craniotomy (Figure 5E). If a tail cannula has not been installed, we next tail vein inject FITC-dextran (7 mg in 0.2 mL lactated Ringer's).

The animal is then moved onto the spherical treadmill located at the two-photon microscope. The head is immobilized by fastening the head bar onto the modified CP02 cage plates (Figures 5F,G). The animal will begin to come out of anesthesia in approximately 5–10 min on the spherical treadmill. If a tail artery cannula was implanted, we load FITC-dextran at this time through the cannula to label vasculature. FITC-dextran will remain in the vasculature for several hours provided that the health of the cranial window is not compromised. The animal can be left to recover and become comfortable on the ball treadmill prior to experimentation. During this recovery time, we typically acquire depth stack images of the window to map the microvascular network. We wait until the mouse displays vigorous running and appears alert (~30 min) before proceeding with experimental trials. Additionally, robust spontaneous diameter changes in arterioles and increased brain blood flow are indicators that the mouse is fully awake.

### Squaring the window with the objective lens

**Figure 6:**
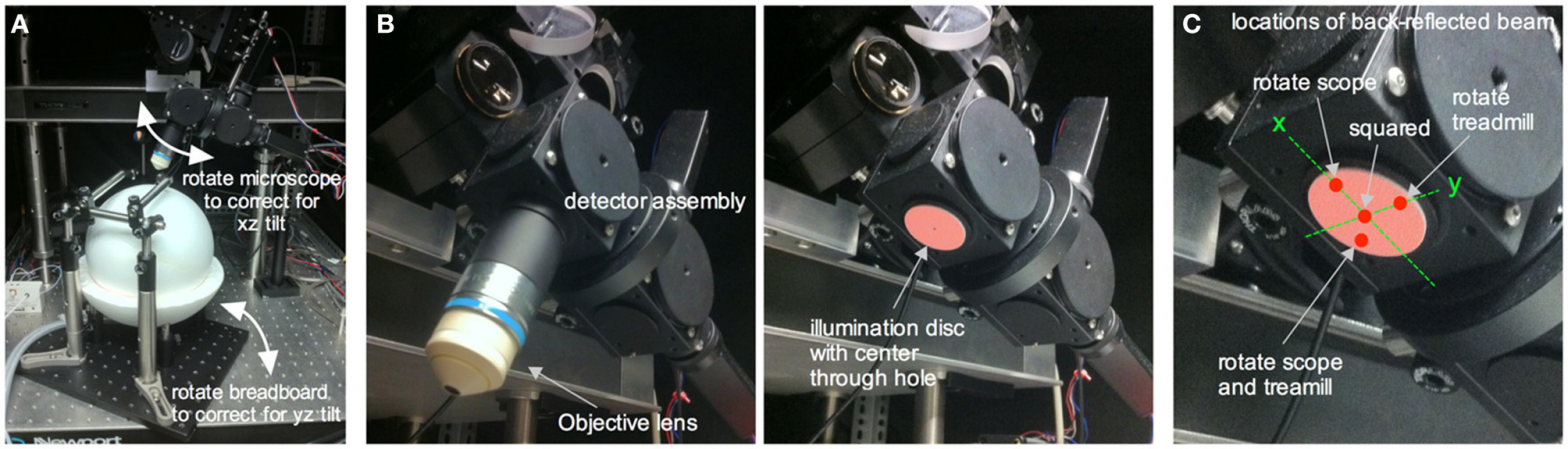
**Squaring an off-axis cranial window to the *in vivo* microscope**. **(A)** The *in vivo* microscope positioned over the ball treadmill in a typical imaging configuration. Rotating the microscope helps correct for xz tilt in the cranial window relative to the optical axis of the microscope. Rotating the entire treadmill apparatus (and thus the head restrained mouse's cranial window) corrects for yz tilt in the cranial window. **(B)** Underside of the detector assembly with the objective lens (left) and with the objective lens replaced by a NIR laser illumination disc containing a center through hole to allow the incoming laser beam to reach the cranial window (right). **(C)** Close up of the illumination disc showing a few different locations of the reflected beam once it has bounced off the cranial window and subsequently illuminated the disc. A spot along the x axis of the disc is corrected for by rotating the *in vivo* microscope. A spot located along the y axis is corrected for by rotating the treadmill apparatus (i.e., the cranial window). A spot in any of the four quadrants requires both x and y correction. After rotational adjustments such that the reflected spot hits the center through hole, the window is square with the optical axis of the microscope and thus to the objective lens.

We use a custom-built two-photon microscope in which the objective lens translates on three linear axes (xyz) and one rotational axis (along the x), similar to the Sutter MOM, Thorlabs Bergamo, or the open-source MIMMS microscope (https://openwiki.janelia.org/). The rotational capability enables imaging of an off-axis cranial window, such as that over the barrel cortex, while keeping the head of the animal in a natural position. This is critical for awake imaging as the head cannot be forced off the level. An off-axis cranial window will deviate from a level horizontal plane in both the xz and yz direction. We adjust the relative positions of the treadmill apparatus (and thus the mouse's cranial window) and the objective lens so that the cover glass of the cranial window becomes perpendicular to the objective lens for optimal imaging (Figure 6A). Though there are measurement based methods available for this (Scheibe et al., 2011), we square the window through an iterative process that aligns the incoming Ti:Sapph beam with the same beam once it has reflected off the cranial window. If the reflected beam deviates at some angle away from the incoming beam, the window is not square. If the incoming beam and the reflected beam travel the exact same path, the window is square to the optical axis of the microscope (!!laser safety goggles are required for the following steps!!). This is tested by replacing the objective lens with a mounted beam illumination disc that has a hole in the center to allow the Ti:Sapph beam to pass (VRC2D1 + modified SM1A7 + adapter collar, Thorlabs) (Figure 6B). An additional hand-held illumination card (VRC5, Thorlabs) is used to ensure the incoming beam strikes the center of the cranial window. The illumination disc facilitates visualization of the beam after it has reflected off the cranial window cover glass back toward the microscope to see the angle of deviation in both the x and y direction. If the reflective beam strikes anywhere on the x-axis of the illumination disc, except at the center hole, then it can be corrected by rotating the microscope (Figure 6C). On the other hand, if the reflective beam strikes anywhere on the y-axis, except at the center hole, then rotating the treadmill apparatus can rectify it. Finally, if the reflective beam does not land on either axis, but instead in one of the four quadrants, then it can be corrected by rotating both the microscope and the treadmill (Figure 6C). These adjustments are made repeatedly until the back reflected beam follows the same light path as the incoming beam i.e., when the reflected beam is at the center hole of the illumination disc.

### Imaging the vasculature in awake mice

**Figure 7:**
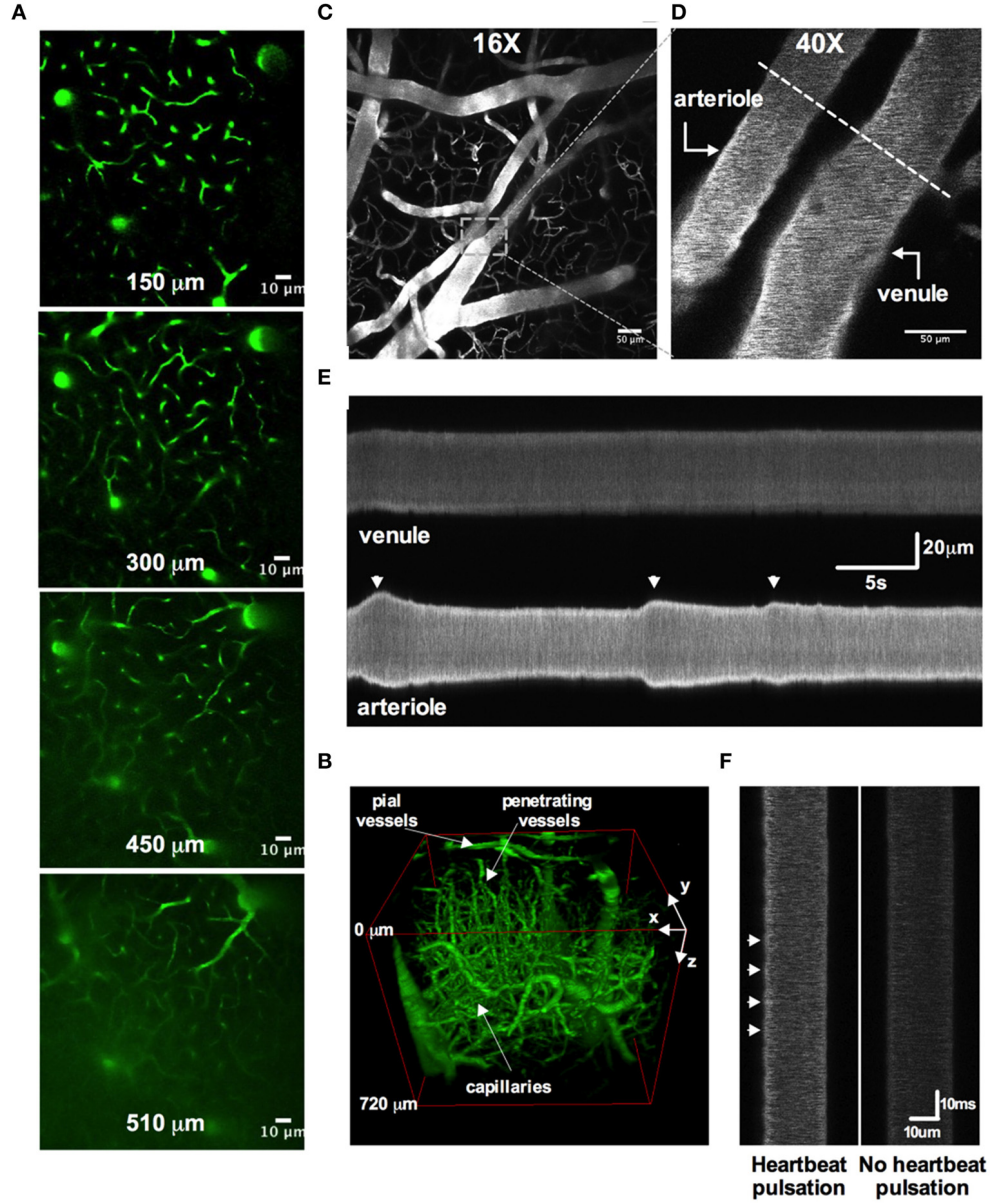
**Two-photon awake imaging of the microvasculature over the barrel cortex**. **(A)** Two-photon fluorescence images at different depths from the surface (150, 300, 450, and 510 μm) as viewed through the acute cranial window. The microvascular network is labeled with FITC-dextran. **(B)** Three-dimensional rendering of the same vascular network z-stack data shown in **(A)**. **(C)** Image of the superficial pial vascular and capillary network. **(D)** Higher magnification image of the same network in **(C)**. A pial arteriole and venule are shown. Dotted line depicts an example of a line scan position across both arteriole and venule. **(E)** Space-time plot of line scan data showing an active pial arteriole showing spontaneous vasomotion, juxtaposed to a quiescent pial venule. **(F)** Line scan data showing heartbeat pulsations along the wall of a pial arteriole (left) and the absence of such deflections in the pial venule (right).

One advantage of a cranial window with bone and dura removal is the ability to image deep within brain tissue. A polished and reinforced thinned-skull window (PoRTS), in which the skull and the dura remain intact, can reach a maximum depth of 250 μm (Shih et al., 2012b) and while there are benefits with the intact nature of this preparation, our open-skull window regularly allowed vascular data acquisition at depths of 500 μm (Figures 7A,B), with a maximal depth of 960 μm (Rosenegger et al., 2014) using a Nikon 16X 0.8NA water immersion objective. This was achieved even when imaging at relatively short wavelengths for two-photon (770 nm).

**Figure 8:**
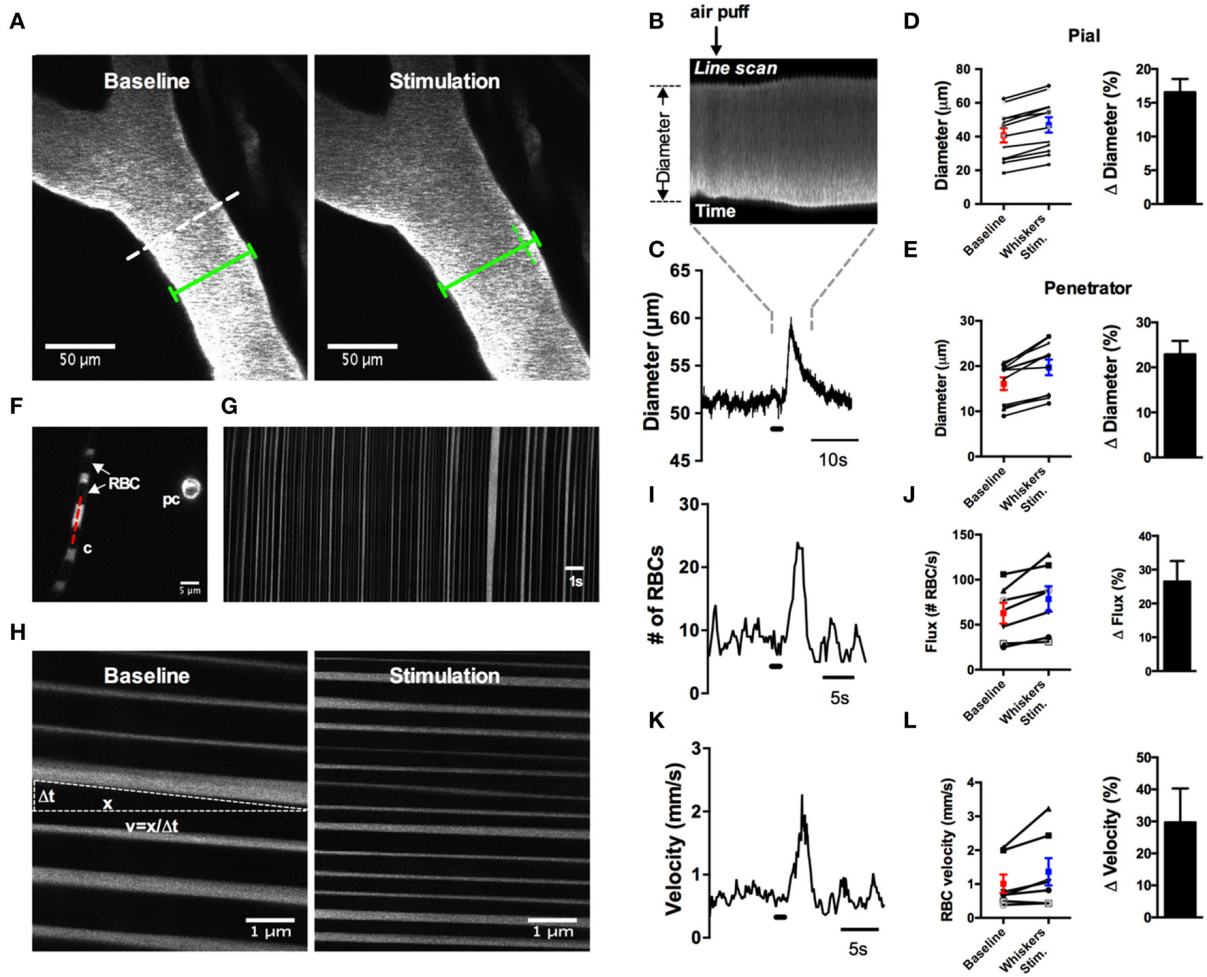
**Whisker stimulation induces robust vasodilation and increases in red blood cell flux and velocity in awake animals**. **(A)** Image of FITC-dextran labeled pial arterioles under basal condition (left) and in response to a 1 s air puff to the contralateral whiskers (right). Dotted line indicates an example line scan path. Green lines help visualize the diameter change. **(B)** Raw line scan data showing arteriole dilation in response to whisker stimulation. **(C)** Line scan data trace from the raw data in **(B)**. **(D)** Left: absolute pial diameter measurements in the absence or presence of whiskers stimulation. Black lines display paired observations of each vessel. Right: Normalized summary data showing percent diameter change in response to whisker stimulation (*n* = 12 animals). **(E)** Similar to **(D)** but the measurements were made from penetrating arterioles (*n* = 11 animals). **(F)** Image of a capillary (c) with visible red blood cells (RBCs) seen as black stripes and a cross section of a pre-capillary (pc). Red dotted line is an example line scan path. **(G)** Space-time plot of line scan data of RBCs passing through a capillary. RBCs appear as dark streaks. **(H)** Space-time plot of line scan data of RBCs under basal condition (left) and in response to whisker stimulation (right). RBC velocity calculated by the distance of travel (x) over the time of travel (Δt). **(I)** Representative trace showing an increase in RBCs flux in response to 1 s air puff to the contralateral whiskers. **(J)** Left: Summary of absolute value of flux measurements. Right: Summary data showing the percent change of sensory-induced flux increases (*n* = 8 animals). **(K)** Representative trace displaying an increase in RBCs velocity to whisker stimulation. **(L)** Left: Calculated RBCs velocity in the absence or presence of stimulation of the whiskers. Black lines represent paired observations from single capillaries. Right: Summary data showing the percent change of sensory-induced RBC velocity increases (*n* = 8 animals).

Several studies have clearly demonstrated how commonly used anesthetics dramatically reduce or abolish various aspects of hemodynamics (Iadecola et al., 1995; Lei et al., 2001; Pisauro et al., 2013). Consistent with previous works, we observed robust spontaneous and evoked changes to the microcirculation in fully awake animals, which were not present as the animal was recovering from isoflurane anesthesia. First, we found that arterioles displayed large magnitude and rapid vasomotion, with no observable changes to venule diameter (Figures 7C–E). Arterioles also exhibited distinct heart beat pulsations that were absent in venules (Figure 7F). Second, using contralateral vibrissae stimulation with air puff to cause functional hyperemia, we measured large diameter changes in pial (16.6% ± 2.0, *n* = 12 mice) and penetrating arterioles (22.9 ± 3.0, *n* = 11 mice) (Figures 8A–E). For each arteriole we made multiple diameter measurements and examined several arterioles per animal. Using line scanning to monitor changes in RBC velocity and flux within capillaries (Figures 8F–H), large increases were detected in response to simulation of the whiskers in awake, active animals (flux 46.2 ± 20.4; velocity 44.4 ± 17.3, *n* = 8, Figures 8I–L). These data indicate a robust and active microvasculature following the acute, awake imaging protocol outlined here.

### Manipulating cerebral microcirculation by intraluminal perfusion via the tail artery

**Figure 9:**
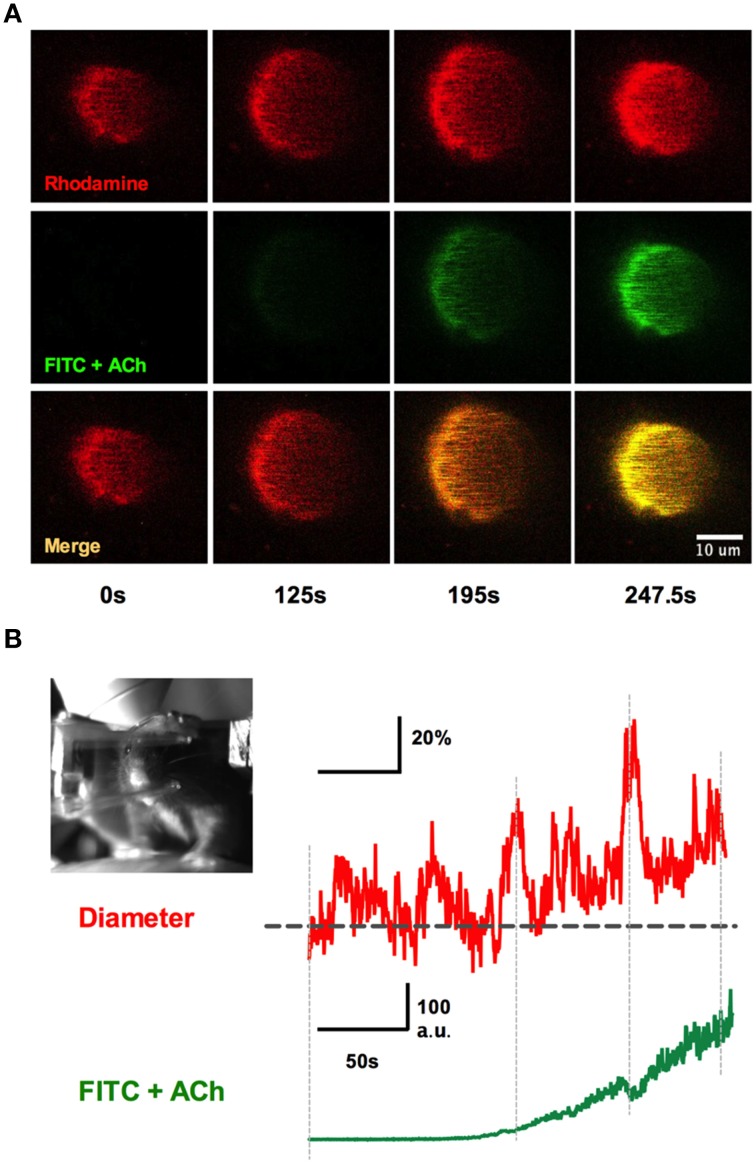
**Systemic infusions via the tail cannula and detection at the window**. **(A)** Rhod-dextran label vasculature before and during the infusion of acetylcholine (ACh) with FITC-dextran through the tail artery cannula while vasomotor responses were monitored. Note the arrival of the green FITC-dextran signal and the increase in arteriole diameter. **(B)** Arteriole diameter trace showing percent change responding to intraluminal perfusion of ACh (top). The bottom trace depicts the rise of FITC-dextran signal in the vasculature, indicating the presence of ACh in the lumen. Inset shows the awake mouse during ACh intraluminal infusion.

We implanted a tail artery cannula to test if we could deliver luminal dyes and pharmacological agents to the brain via the vasculature during two-photon imaging in awake animals when using a fully sealed cranial window (Figure 9). We first loaded Rhod-dextran to visualize brain microvasculature within the window. Acetylcholine (ACh) acts on M3 muscarinic ACh receptors on endothelial cells to cause vasodilation (Emerson and Segal, 2000; Domeier and Segal, 2007; Tran et al., 2009; Butcher et al., 2013). We delivered ACh through the cannula using a standard syringe pump (syringe concentration 100 μM in 250 μL Ringer's perfused at 10 μL/min) during an imaging sequence of a single penetrating arteriole to specifically activate the endothelium. 0.2 mg of FITC-dextran was also included in the perfused solution so that we could approximate the arrival of ACh to the brain. We found that the detection of green FITC fluorescence in the lumen of the vessel was well timed with an increase in arteriole diameter (Figures 9A,B). These data suggest that the time in which intravascularly delivered compounds arrive to the brain can be visualized, in order to study pharmacological manipulations using a fully sealed cranial window. Depending on the experiment, blood brain barrier permeable or impermeable agents can be employed to target brain cells, or endothelial cells respectively, when combined with a large molecular weight luminal dye.

### Simultaneous two-photon imaging with behavior capture

**Figure 10:**
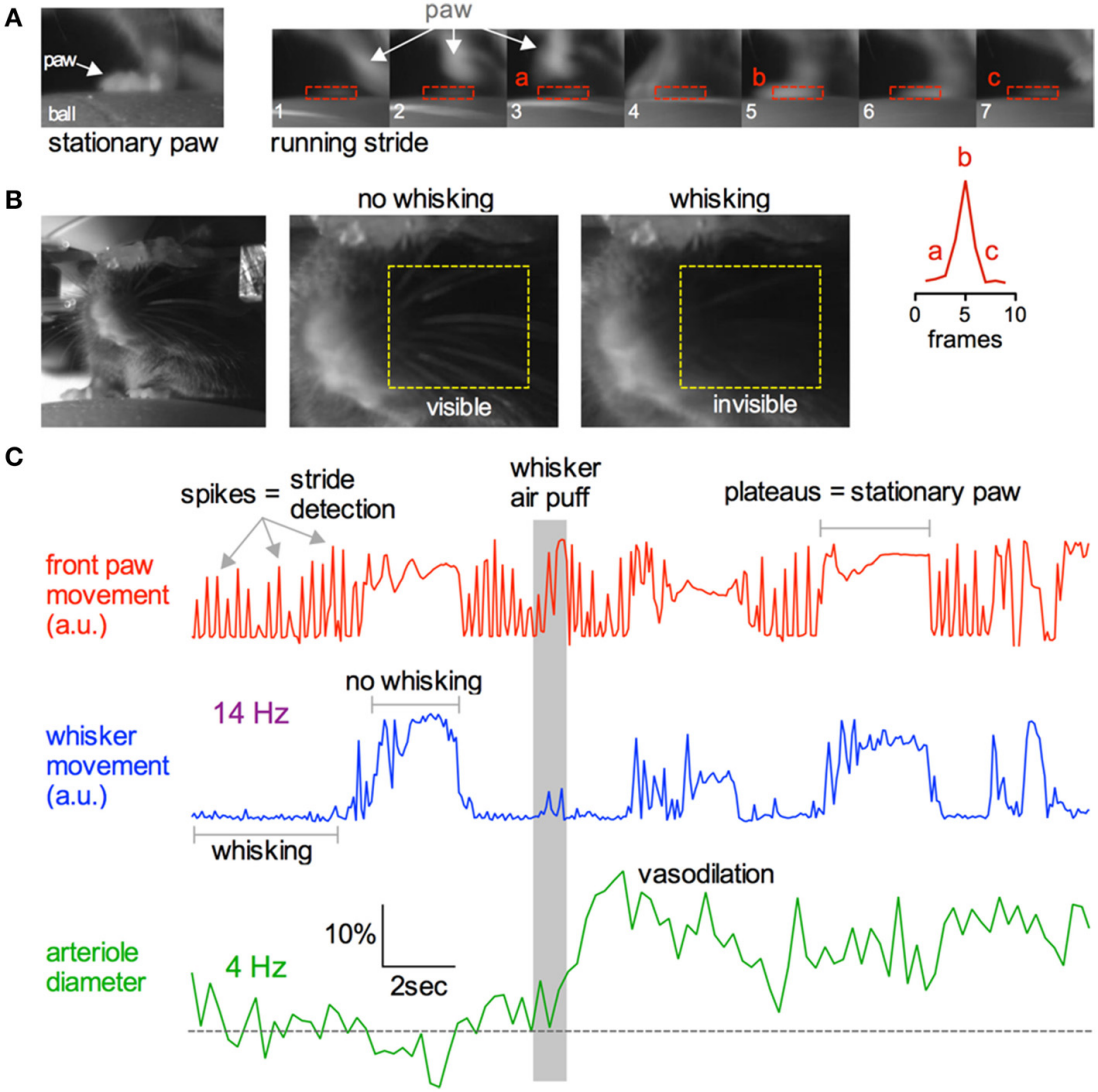
**Capturing running and whisking behavior simultaneously with two-photon imaging**. **(A)** Near infrared camera image of a stationary right front paw on the ball treadmill (left) and a sequence of images depicting the same paw during a running stride (right). Lower right inset graph demonstrates that each running stride results in a spike in signal intensity as the paw enters and leaves the ROI. **(B)** Head restrained mouse resting on the treadmill (left). Images showing how the behavioral camera can be used to detect whisking vs. not whisking. Stationary whiskers reflect the near infrared light source toward the camera creating higher raw gray values. Whisking reduces this reflectance, dropping the gray value. **(C)** Example traces showing simultaneous collection of front paw movement (top red), whisking (middle blue), and two-photon fluorescence capture of arteriole diameter (bottom green). Gray bar indicates time of whisker air puff, resulting in sensory evoked vasodilation.

Being able to simultaneously monitor animal behavior and physiological responses at the cellular level is a great advantage of awake *in vivo* two-photon imaging. We tested if we could capture simple behaviors such as resting, running or whisking using a near infrared camera simultaneously with two-photon fluorescence data (Figure 10). As a mouse ran on the ball, its running stride could be detected by creating a region of interest (ROI) around the right forepaw and by measuring the pixel gray value as the paw moved in and out of the ROI (Figure 10A). Each stride generated a spike in the gray value, while a plateau in the gray value signified a stationary paw (Figures 10A,C). Similarly, a ROI could be placed over a region of whiskers to detect the presence or absence of whisking. We found that stationary whiskers reflected the near infrared light source toward the camera showing a high gray value, while whisking decreased the reflectance and thus reduced the gray value (Figures 10B,C). These measurements could be made simultaneously with two-photon imaging of arteriole diameter changes caused by vibrissae stimulation with air puff (Figure 10C).

**Figure 11:**
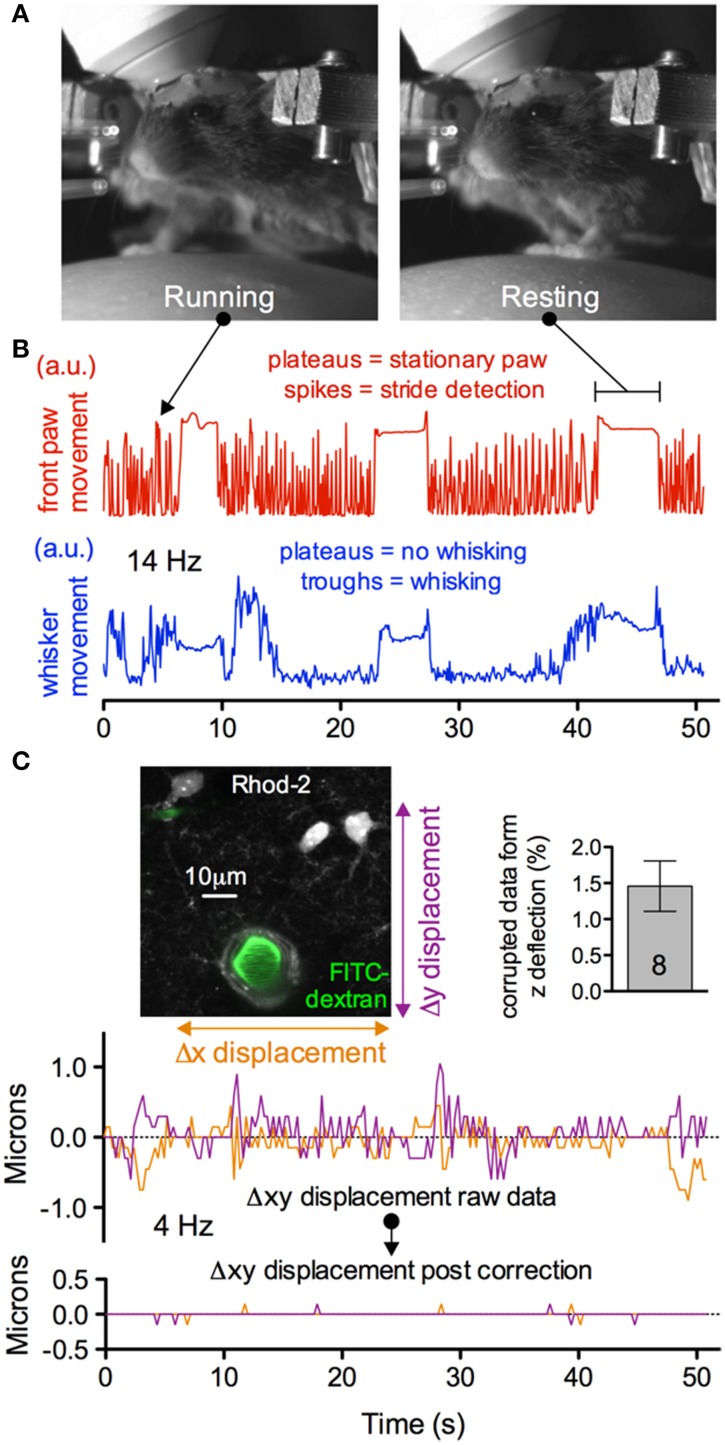
**Subcellular two-photon imaging in active mice with acceptable movement artifacts**. **(A)** Near infrared camera image of an active, head restrained mouse showing running (left) and resting (right). **(B)** Upper trace showing right front paw stride detection during running (spikes) and stationary paw during resting (plateaus). Lower trace showing the detection of whisking (troughs) and no whisking (plateaus) from the same experimental trial. **(C)** Motion artifacts in the x and y plane detected during the trial shown in **(B)**. Image shows high magnification field of view of astrocytes, endfeet and a penetrating arteriole (left). Summary data showing a low percentage of astrocyte somata and endfeet data lost due to motion in the z direction during behavior (right). Traces show the raw (upper) and corrected (lower) x and y deflections in the image sequence during the awake mouse activity displayed in **(B)**. Movement artifacts in the x and y plane can be corrected *post hoc*.

A concern with fully awake animal two-photon imaging is the presence of significant movement artifacts. Installation of the head bar for skull immobilization is essential to mitigate motion in recorded images. Nevertheless, as the animal runs artifacts are generated (Figures 11A–C). However, we found that when imaging astrocytes, endfeet and penetrating arterioles at 4 Hz at high magnification, translational movements in the x and y direction could be well corrected using standard frame-by-frame algorithms (Figure 11C). This may be improved further by line-by-line algorithms that correct for distortions within a single frame (Dombeck et al., 2007). Furthermore, the percent of data lost caused by z deflections of approximately 3 microns or greater was under 2% when imaging astrocyte somata and endfeet (8 animals, 3 image sequences from each) (Figure 11C insert). Collectively, these data demonstrate the ability to measure running and whisking behavior while making observations at the cellular level in awake and behaving animals with acceptable movement artifacts.

### Imaging astrocytic Ca^2+^ dynamics in awake animals

**Figure 12:**
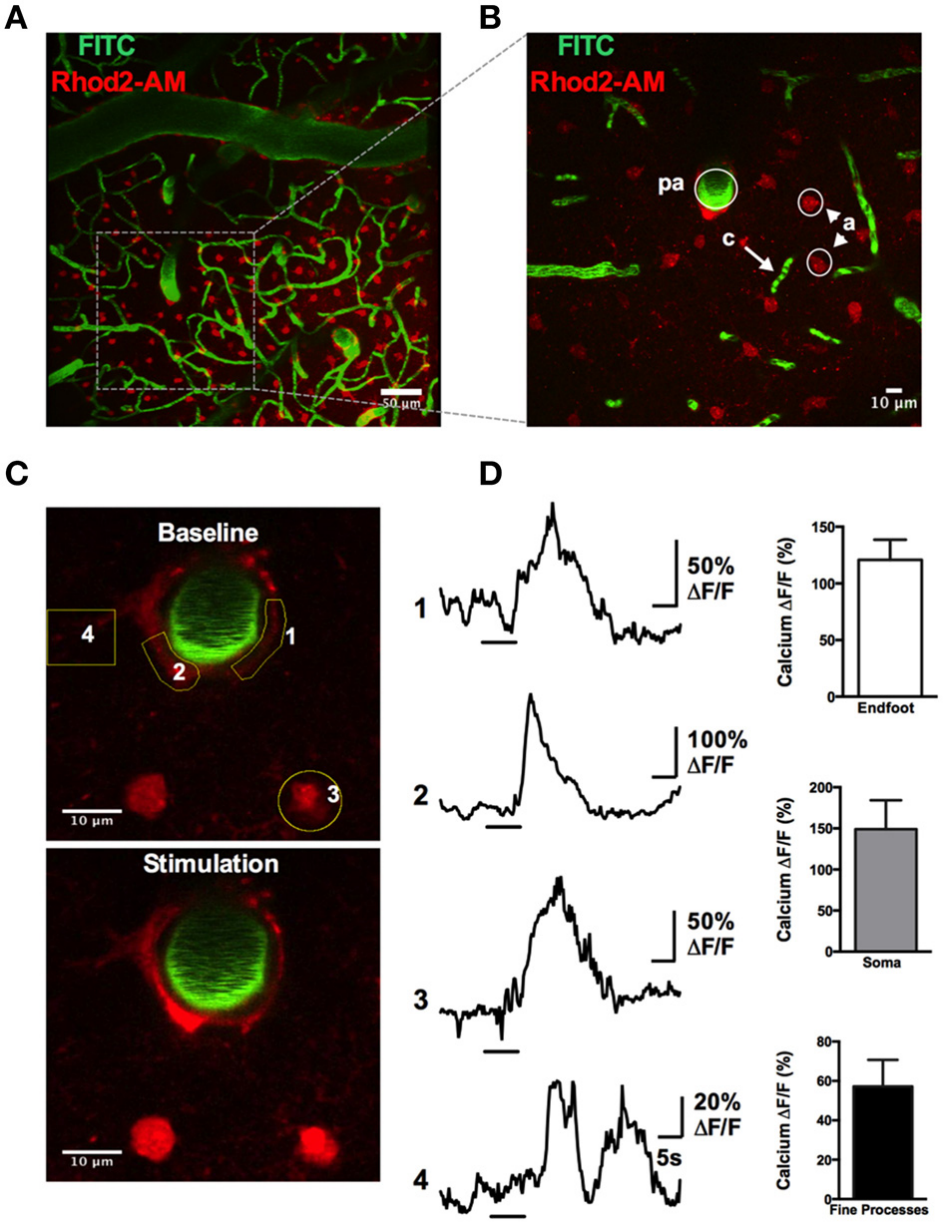
**Astrocytic Ca^2+^ signals observed with the synthetic indicator Rhod2-AM**. **(A)** max-projection Z-stack image showing the vascular network labeled with FITC-dextran (green) and astrocytes loaded with Rhod2-AM (red). **(B)** Higher magnification Z-stack from a region in **(A)**, showing a penetrating arteriole (pa), capillaries (c), and astrocytes (a). **(C)** Images of astrocytic somata and enfeet loaded with Rhod2-AM (red) and the penetrating arteriole labeled with FITC-dextran (green) under basal condition (top) and in response to whisker stimulation (bottom). **(D)** Left: Traces of Ca^2+^ transients following sensory stimulation observed in endfoot (1, 2), soma (3), and fine processes (4). Yellow regions of interest indicated. Right: Summary data showing percent change of sensory-evoked Ca^2+^ obtained from endfeet, somata, and fine processes in responding astrocytes (*n* = 3 animals).

**Figure 13:**
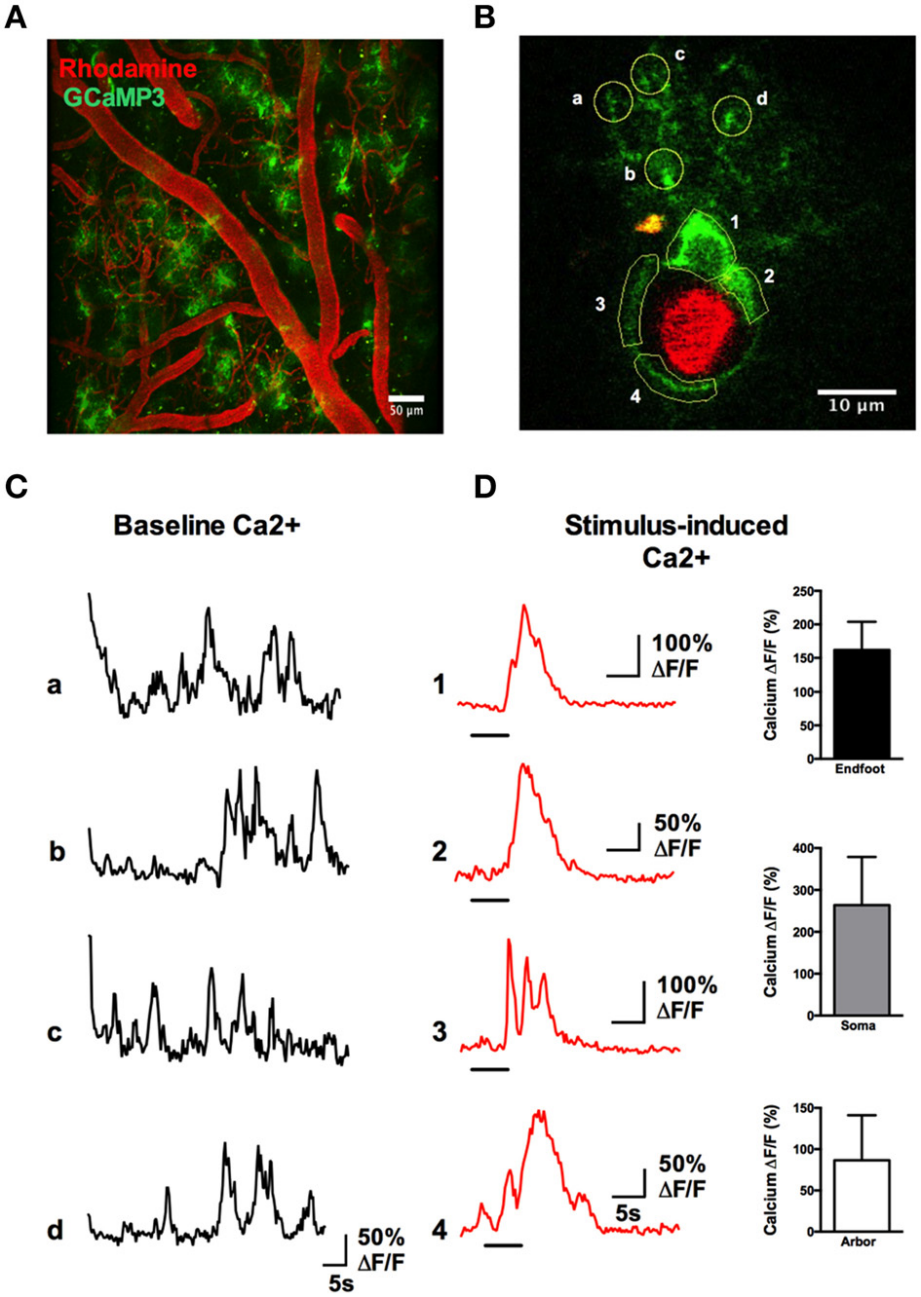
**Astrocytic Ca^2+^ signals observed with the genetically encoded indicator GCaMP3**. **(A)** Two-photon fluorescence image showing the vasculature network labeled with Rhod-dextran (red) and GCaMP3 expressed in astrocytes (green). **(B)** A single astrocyte wrapping a penetrating arteriole, with regions of interest indicated. **(C)** Traces of spontaneous Ca^2+^ transients detected in the astrocyte arbor from letter regions in **(B)** in the absence of whisker stimulation. **(D)** Left: Traces of Ca^2+^ transients in different astrocyte compartments from the numbered regions in **(B)**, in response to stimulation of the whiskers by 5 s air puff. Right: Summary data showing percent change of sensory-evoked Ca^2+^ obtained from endfeet, somata, and fine processes in responding astrocytes (*n* = 3 animals).

A rapid elevation in astrocyte free intracellular Ca^2+^ is used as an index of the cell's activity (Cornell-Bell et al., 1990). Such astrocyte Ca^2+^ transients have recently been observed to be dramatically attenuated by anesthesia (Thrane et al., 2012). Consistent with this, the acute, awake imaging protocol executed here allowed us to detect frequent and robust Ca^2+^ transients in astrocytes in active mice. Using the Ca^2+^ indicator Rhod2-AM, astrocyte somata, endfeet and FITC-dextran labeled vasculature were clearly visualized in the cranial window (Figures 12A,B). Large magnitude Ca^2+^ signals from subcellular compartment were observed in some trials in response to vibrissae stimulation (ΔF/F endfoot: 120.9 ± 17.8%; soma: 149.1 ± 35.2%; arbor: 57.2 ± 13.5%, *n* = 3 mice, Figures 12C,D). Next, we tested our protocol for use of conditional Cre-Lox mice in which only astrocytes expressed the genetically encoded Ca^2+^ indicator GCaMP3 using the GLAST promoter (Figures 13A,B). GCaMP3 exhibits low baseline fluorescence at resting levels of Ca^2+^ (Zariwala et al., 2012; Shigetomi et al., 2013), but showed large percent changes in spontaneous Ca^2+^ signals occurring in astrocyte arbors (not experimentally evoked). As well, Ca^2+^ signals were observed in all astrocyte compartments in response to vibrissae stimulation in some trials (ΔF/F endfoot: 161.9 ± 21%; soma: 263.8 ± 51.4%; arbor: 86.5 ± 24.5%, *n* = 3 mice, Figures 13C,D). Our ability to detect basal GCaMP3 fluorescence at 4 Hz was undoubtedly aided by the window being free of bone and dura for optimal excitation and fluorescence collection. These data show that our protocol enables subcellular measurements of astrocyte Ca^2+^ activity in awake behaving animals.

## Discussion

Awake animal imaging is a key advance in order to observe non-confounded physiological processes. For the neurosciences, there are obvious advantages in being able to make connections between sensory activity, motor output, or learning to cellular events in the brains of awake animals. However, imaging awake animals has several challenges. First, head immobilization is a necessity for standard two-photon imaging at the subcellular level, which is a stressor for rodents (Schwarz et al., 2010). The protocol outlined here was to establish an effective method that would minimize stress and struggling against head-fixation, while at the same time being efficient and productive. Qualitatively comparing siting in a tube, to a linear passive treadmill, to a spherical passive treadmill, we found that the air supported ball was superior in terms of minimizing struggling, squeaking and learned helplessness. Animals began running immediately upon being placed on the ball, and it was only their adeptness and control of the ball that was initially poor, but this was significantly improved by the imaging session. Being familiar with the experimenter can help reduce stress in mice during behavioral experiments (Dombeck et al., 2007). However, we found that an initial handling session of a C57Bl/6 mouse before the first head fixed session failed to clearly reduce stress behaviors compared to having no initial handling. This was likely because experimenter familiarization is a minor component of overall stress compared to head immobilization. For this reason, we largely eliminated animal handling, and practiced swift transfers from cage to ball, and ball to cage. This relieves the experimenter of an extra training session. We also found that head-fixed training sessions in excess of two were met with little behavioral improvement. This allowed proportionally more rest days for the animal while keeping with a 5-day workweek. Finally, our training procedure does not require rewards, which may be useful in studies in which goal and reward neural circuitry needs to be carefully considered for the experiment itself, rather than for training for the experiment.

The protocol presented here uses bone and dura removal. Alternatively, removing the bone but keeping the dura intact has positives and negatives. In the positive, intact dura keeps the brain from swelling out of the craniectomy. Thus, there is less need for pre dexamethasone treatment. In the negative, dura reduces maximal depth penetration, especially when large dura blood vessels are present directly above the imaging field. Furthermore, the dura can be a significant source of bleeding. When using luminal FITC or Rhodamine-dextran to visualize microvasculature diameter and red blood cell movements, damaged vessels in the dura will leak the dye, quickly ruining an imaging session. The technique described here is amenable to thinned skull preparations such as PoRTS (Shih et al., 2012a). Imaging through a cranial window with bone removal, is associated with substantial microglia and astrocyte activation from 24 h to 1 month after surgery, as well as altered physiology (Xu et al., 2007; Grutzendler et al., 2011). Less invasive protocols, such as bone thinning, are thought to minimize inflammation and provide a more realistic condition, however, the depth and clarity of the observations are limited to the first one to two hundred microns of the tissue (Shih et al., 2012a) and the capture of signals from weak fluorophores would be difficult.

The barrage of signals coming from multiple levels within the nervous system presents a complex environment and a significant challenge to understand the data collected from awake and behaving animals. Increased inter and intra experimental variance will likely necessitate more trials and animals to clearly delineate effects. However, effects that are recorded are less likely to be met with skepticism on the veracity or trueness of the phenomenon, given the realistic conditions of the experiment. Linking observations on the cellular level with behavior is an important aspect for awake animal imaging. The setup shown here provides superficial assessment of whisking and running behavior, but more detailed behavioral analysis can be implemented following procedures outlined elsewhere that use purpose-selected hardware and software. For instance, x and y directional distances and accelerations can be measured from the surface of the ball treadmill using optical computer mice to track ball motion (Dombeck et al., 2007). A more detailed and careful analysis of whisker movements could also be achieved with whisker trimming, appropriate back lighting and using whisker tracker hardware and software (O'Connor et al., 2010). To achieve more natural behaviors simultaneously with imaging data, an alternative to head-restraint is to employ miniaturized head-mountable microscopes (Helmchen et al., 2001; Kerr and Nimmerjahn, 2012). For imaging neurons and microvasculature in freely moving animals, this principle has been demonstrated (Helmchen et al., 2001). However, the temporal and spatial resolution is not as high as standard two-photon imaging. The procedure outlined here is more practical and can be adapted onto many existing two-photon systems if adequate space under the objective can be achieved.

Imaging awake animals is increasingly capturing the interest of many neuroscience fields, especially neurovascular coupling for the dramatic effects of anesthetics on hemodynamics (Iadecola et al., 1995; Pisauro et al., 2013). Anesthetized preparations used for brain blood flow research require intubation and a ventilator to maintain appropriate blood gases and blood pH, coupled with regular monitoring of these parameters via blood samples. Awake preparations largely eliminate this need as the animals self regulate pO2 and pCO2. However, an assessment of general brain perfusion on the pial surface and/or blood pressure would be advantageous, as a small fraction of animals do not show robust recovery from the surgery. Because anesthetics eliminate perception, they provide a platform to test and compare experimental manipulations using invasive procedures. However, anesthetics impose numerous effects on brain activity and hemodynamic measures, placing a significant confound to any data acquired during their use. For instance, synaptic transmission is a primary driver of neurovascular coupling, yet altered synaptic transmission is thought to be a main site of action of general anesthetics. Important second messengers such as cAMP and cGMP are altered by ketamine, pentobarbital and halothane (Kebabian et al., 1975; Lenox et al., 1979). Additional effects on nitric oxide synthase (Tobin et al., 1994) suggest that the essential vasodilator nitric oxide is also impacted. As nitric oxide derives from both vascular endothelium and activation of neural NMDA receptors, this is a large confounding factor for studies on neurovascular coupling (Iadecola et al., 1995; Pisauro et al., 2013). The effect on blunted NMDA receptor activation can be attributed to Ketamine (Erchova et al., 2002) while other anesthetics can affect glutamate release itself (Haseneder et al., 2004). Furthermore, at least one mode of action of isoflurane and α-chloralose is to potentiate GABA_A_ receptor chloride currents and thus decrease excitability (Garrett and Gan, 1998; Eikermann et al., 2011; Rivadulla et al., 2011). Consistent with this action, isoflurane has been demonstrated to reduce the amplitude of the hemodynamic response and also delay the onset (Pisauro et al., 2013). One of the most commonly used anesthetics for animal studies is urethane because of its deemed minimal effect on the cardiovascular system. However, urethane does affect a variety of neurotransmitter-gated ion channels (Hara and Harris, 2002), making it difficult to tease apart real physiological phenomenon in neurovascular coupling vs. the side effects of anesthesia. Furthermore, a study by Thrane et al. (2012), showed that even low levels of anesthesia, which failed to affect neuronal responses to whisker stimulation, decreased astrocyte Ca^2+^ signals. These studies highlight the need for more work in awake animals to help clarify these physiological signals. It was our aim with this protocol to aid laboratories in their efforts to better understand brain physiology under realistic conditions.

### Conflict of interest statement

The authors declare that the research was conducted in the absence of any commercial or financial relationships that could be construed as a potential conflict of interest.
